# Characteristic amino acid residues in the growth hormone receptor gene on *Mus minutoides* underlying dwarfism

**DOI:** 10.17912/micropub.biology.000955

**Published:** 2023-09-11

**Authors:** Sumito Matsuya, Kaoru Fujino, Hiroyuki Imai, Ken Takeshi Kusakabe, Kiyoshi Kano

**Affiliations:** 1 Laboratory of Developmental Genetics, The United Graduate School of Veterinary Science, Yamaguchi University, Yamaguchi, Yamaguchi, Japan; 2 Laboratory of Veterinary Anatomy, Joint Faculty of Veterinary Medicine , Kagoshima University, Kagoshima, Kagoshima, Japan; 3 Laboratory of Developmental Genetics, Joint Faculty of Veterinary Medicine, Yamaguchi University, Yamaguchi, Yamaguchi, Japan; 4 Laboratory of Veterinary Anatomy, Joint Faculty of Veterinary Medicine, Yamaguchi University, Yamaguchi, Yamaguchi, Japan

## Abstract

The African pygmy mouse ( *Mus minutoides* ) displays a dwarfism phenotype distinctive from closely related species. This study aimed to investigate the growth hormone receptor (Ghr) gene sequence in *M. minutoides* . We identified several amino acid variations, including the P469L mutation. Our findings suggest that this mutation affects Ghr protein functionality, decreasing *Igf1* expression and contributing to the dwarfism observed in *M. minutoides* . Further studies utilizing genome editing technology are necessary to elucidate the mechanisms involved in mammalian body size determination.

**Figure 1. Growth hormone receptor (Ghr) sequence analysis.**
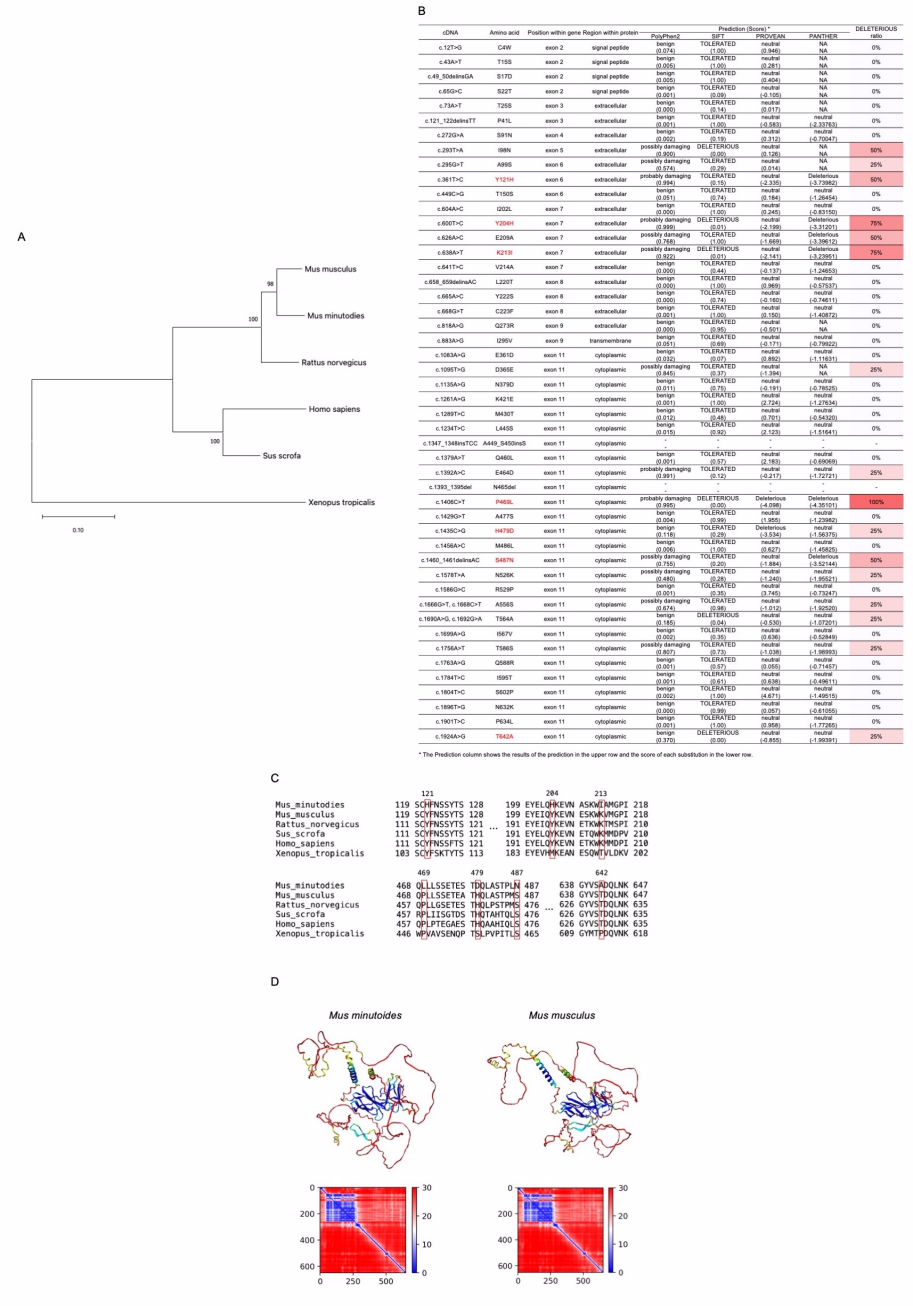
(A) Phylogenetic analysis of Ghr sequences using the neighbor-joining method. Bootstrap values are presented at each node, and the evolutionary distance is represented by a straight line in the lower left corner. (B) Amino acid variation of Ghr in *Mus minutoides* and the evaluation scores by VaProS. Amino acid variations in red letters indicate amino acid residues shown in Figure 1C. (C) Partial alignment analysis of the predicted amino acid sequences of Ghr. The numbers indicate the corresponding amino acid positions, and the boxes highlight the regions where the amino acid residues differed between *M. minutoides* and *M. musculus* . (D) (Upper) Structural models of Ghr for *M. minutoides* and *M. musculus* generated using AlphaFold2. (Lower) Predicted Aligned Error (PAE) depicting the reliability of positional relationships between residues for each structural model.

## Description

The size of mammals varies from mice to whales and can vary markedly from species to species within the same genus. *Mus minutoides* is recognized as one of the smallest mammals in the world. An adult typically weighs approximately 3 g and is approximately 30 mm long. It belongs to the same genus as *Mus musculus* , a well-known laboratory animal, but is tenfold lighter (Willan and Meester, 1978) . Despite the physiological similarities shared by the two species, the molecular mechanisms responsible for the notable differences in body size observed between these two species and across a wide range of mammals remain poorly understood.

The Gh-Igf1 axis plays a role in regulating body and organ size (Vasques *et al.* , 2019). It has been extensively studied in mice, humans, dogs, and cows, focusing on its function and mutations (Godowski *et al.* , 1989; Chen *et al.* , 1991; Lupu *et al.* , 2001; Iio *et al.* , 2020). The growth hormone receptor gene ( *Ghr* ), a key component of the Gh-Igf1 axis, is expressed primarily in the liver and is responsible for stimulating insulin-like growth factor 1 (Igf1) production in response to growth hormone (Gh) secreted by the anterior pituitary gland (Vasques *et al.* , 2019). Igf1 is crucial in promoting growth by stimulating cell division and metabolism throughout the body (Wang *et al.* , 2004; Yakar *et al.* , 2018). Laron syndrome (LS) is a form of human dwarfism associated with the Gh-Igf1 axis (Godowski *et al.* , 1989; Schaefer *et al.* , 1994; Iwatani *et al.* , 1997; Janecka *et al.* , 2016). It is caused by various genetic mutations, such as exon loss or mutations, in *Ghr* . Several reports have documented dwarfism resulting from defective Igf1 secretion due to Ghr dysfunction (Rosenbloom and Guevara-Aguirre, 1998; Werner *et al.* , 2020). *Ghr* knockout experiments in mice and pigs have successfully replicated the LS phenotype (Zhou *et al.* , 1997; Cui *et al.* , 2015), emphasizing the crucial role of Ghr in determining mammalian body size. Based on these findings, we propose that *Ghr* may be involved in the molecular mechanisms underlying dwarfism in *M. minutoides* .

*M. minutoides* and *M. musculus* formed the most closely related cluster within the order Rodentia, family Muridae ( Figure 1A ). In contrast, *Homo sapiens* and *Sus scrofa* formed a distinct cluster separate from the Rodentia group. Additionally, a detailed alignment analysis was conducted using CLUSTALW. The predicted length of the *M. minutoides* Ghr protein was 650 amino acids, which is equivalent to the length of *M. musculus* Ghr. However, 48 amino acid residues (7.38%) differed between *M. minutoides* and *M. musculus* Ghr ( Figure 1B ). To assess the functional implications of the amino acid differences in *M. minutoides* Ghr, we utilized VaProS (http://p4d-info.nig.ac.jp/vapros/). Four algorithms were employed, namely PolyPhen2, SIFT, PROVEAN, and PANTHER (cutoffs = 0.5, 0.05, -2.5, and -3, respectively). Amino acid substitutions that scored above the cutoff for PolyPhen2 or below the cutoff for SIFT, PROVEAN, and PANTHER were considered to have a negative effect. As a result, it was found that in all four algorithms, no significant impact on the protein's function was observed for 30 amino acid residues, while 16 amino acid residues were predicted to have the potential for functional impairment ( Figure 1B ). Insertions and deletions (A449_S450insS and N465del) could not be evaluated by these algorithms, as they are specifically designed to assess only amino acid substitutions.

Furthermore, an alignment analysis was performed on six animal species, including *Xenopus tropicalis* , which was used as an outgroup for the phylogenetic analysis. We identified seven regions that exhibited high conservation among the species but showed variations in amino acid residues between *M. minutoides* and *M. musculus* (red letters in Figure 1B and red square in Figure 1C). Among the compared Ghr sequences, the amino acid residues Y204H, K213I, H479D, and T642A were identical in *M. musculus* , *Rattus norvegicus* , *S. scrofa* , and *H. sapiens* but not in *M. minutoides* ( Figure 1C ). These findings indicate their significance in mammals. In contrast, amino acid residues Y121H, P469L, and S487N differed exclusively in *M. minutoides* . These unique amino acid residues in *M. minutoides (X. tropicalis* displayed identical residues to those of the other mammals) may contribute to the *M. minutoides* -specific phenotype. Specifically, P469L was predicted to be a "deleterious mutation" by all four algorithms in the variation effect analysis using VaProS. This suggests that amino acid residue changes in *M. minutoides* may significantly affect Ghr protein function. In the context of structure prediction using AlphaFold2, no significant differences between the structures of the Ghr proteins in *M. minutoides* and *M. musculus* were observed ( Figure 1D ). However, proline, the 469th and 458th amino acid in *M. musculus* and *H. sapiens* , respectively, is located in the intracellular domain of the Ghr protein and is crucial for transmitting signals received from Gh into the cell (Smith *et al.* , 1989; Lin *et al.* , 2018). Also, Rowland *et al.* (2005) reported that the amino acid sequence between positions 391 and 569 of the Ghr protein plays an important role in intracellular signal transduction. In most animals, this proline residue is highly conserved, suggesting the conservation of Ghr's intrinsic function across these species. Therefore, the alteration of *M. minutoides* Ghr from proline to leucine (P469L) may significantly modify signal transduction from the receptor to the cell. Furthermore, given that the intracellular domain encompasses a significant portion of the identified amino acid substitutions in *M. minutoides* , it is plausible that the combined effect of these 25 substitutions, including P469L, H479D, S487N, and T642A, may collectively impact its functionality. Our preliminary data showed that *Igf1* expression in *M. minutoides* was significantly lower than in *M. musculus* . Taken together, these findings suggest that the altered amino acid sequence of *M. minutoides* Ghr may hinder intracellular signaling, resulting in decreased *Igf1* expression, and thus contribute to dwarfing in *M. minutoides* .

In conclusion, our study has shed light on novel aspects of Ghr, a crucial component of the Gh-Igf1 axis that plays a significant role in mammalian growth. Although previous studies have examined protein function through amino acid substitutions and deletions in various mammalian Ghr proteins (Goujon *et al.* , 1994; Wang *et al.* , 1995; Vairamani *et al.* , 2017), investigations of various mutations are lacking. We believe that investigating specific functional alterations in Ghr resulting from these mutations using genome editing technology will enhance our understanding of the mechanisms governing mammalian body size determination.

## Methods

This study investigated the characteristics of the *Ghr* gene sequence and explored the relationship between these characteristics and dwarfism in *M. minutoides* . All animal experiments were approved by the Yamaguchi University Animal Use and Care Committee (approval number:291). Total RNA was extracted from the livers of *M. minutoides* using ISOGEN II (NIPPON GENE, Tokyo, Japan). The extracted RNA was reverse transcribed into cDNA using the QuantiTect Reverse Transcription Kit (QIAGEN, Hilden, Germany). Subsequently, primers were designed based on the highly conserved regions among *M. musculus* , *Mus pahari* , and *Mus spretus* using the coding sequences in the *Ghr* gene database. The primer sequences are listed below. PCR amplification was performed using *M. minutoides* cDNA as the template with BIOTAQ DNA polymerase (NIPPON Genetics, Tokyo, Japan) under the following conditions: initial denaturation at 94°C for 3 min, followed by 35 cycles of denaturation at 94°C for 30 sec, annealing at 64°C for 45 sec, and extension at 72°C for 45 sec, with a final extension at 72°C for 1 min. After electrophoresis, the target DNA bands were excised from the agarose gel, and the DNA fragments were extracted using the FastGene Gel/PCR Extraction Kit (NIPPON Genetics). DNA sequencing of the extracted DNA was outsourced to the Yamaguchi University Center for Gene Research. Additionally, the nucleotide sequence was determined using three individuals of *M. minutoides* , and the sequence alignment confirmed their identity (Accession number for the CDS of *M. minutoides* Ghr in DDBJ is LC623619). A phylogenetic analysis was conducted using MEGA X software (Kumar *et al.* , 2018) based on the amino acid sequence predicted from the determined nucleotide sequence of *M. minutoides* . Additionally, the prediction of the protein's structure was performed using the putative amino acid sequence with AlphaFold2 (Jumper et al., 2021; Varadi et al., 2022) .

**Table:** 

Primer name	Forward (5' → 3')	Reverse (5' → 3')
*Ghr* -1	CAGGTCTTCTTAACCTTGGCACTGG	CAGTTGGTCTGTGCTCACATAACCAC
*Ghr* -2	GCCTCGATTCACCAAGTGTCGTTC	ACCACCTGCTGGTGTAATGTCGC
*Ghr* -3	ACTGGCAAAGGCGGCTGCTAC	GGAACGACACTTGGTGAATCGAGGC
*Ghr* -4	TCACACCGTGCAGTCTCCAAG	*GGCCACGCCTCGACTAGTAC Adapter sequences in the 3' RACE method, not present in the Ghr sequence.
